# Supplementary material for: BRCA1/2 Molecular Assay for Ovarian Cancer Patients: A Survey through Italian Departments of Oncology and Molecular and Genomic Diagnostic Laboratories
Source: Diagnostics (Basel). 2019 Oct 9;9(4):146. doi: 10.3390/diagnostics9040146 (PMC6963957; doi:10.3390/diagnostics9040146)
Supplement: Supplementary file 1 [file diagnostics-09-00146-s001.zip › diagnostics-573360-Supplemental File/diagnostics-573360-Supplemental File1.pdf]

SURVEY FOR ITALIAN ONCOLOGY CENTRES:
BRCA AND MANAGEMENT OF THE PATIENT WITH OVARIAN CANCER

1. Is there a genetic counseling clinic in your centre?
   - Yes
   - No

2. Is it formally recognized by the host institution and/or the Region?
   - Yes
   - No

3. Who is responsible for the management of the company?
   - Department of Medical Genetics
   - Department of Oncology
   - Other *(specify)*

4. Who is in the genetic counseling clinic?
   (multiple answers possible)
   - Geneticist Doctor
   - Geneticist Biologist
   - Oncologist
   - Surgeon
   - Nurse
   - Psychologist
   - Other *(specify)*

5. Are the evaluations on **BRCA GERMLINE tests** carried out <u>after</u> evaluation of the ovarian cancer patient in the genetic counseling clinic?
   - YES
   - NO

6. If the answer is NO: is the request made directly by the oncologist (doctor, gynaecologist) after an interview with the patient (*minicounseling*)?
   - YES
   - NO

7. Are the evaluations on **BRCA SOMATIC tests** carried out <u>after</u> the evaluation of the patient in the genetic counseling clinic?
   - YES
   - NO

8. If the answer is NO: is the request made directly by the oncologist (doctor, gynaecologist) after an interview with the patient (*minicounseling*)?
   - YES
   - NO

9. In your CENTRE, in the patient with ovarian cancer, it is carried out as FIRST evaluation:
   - Germline BRCA
   - Somatic BRCA

10. In case BRCA germline test firstly evaluated: if the patient with ovarian cancer is BRCA NOT mutated, do you then perform somatic BRCA?
    - YES
    - NO

11. If the first evaluation is the somatic BRCA test and the patient is found to be mutated, do you perform BRCA germlines for the detection of that mutation?
    - YES
    - NO

12. In the case of a patient with BRCA-germline mutated ovarian carcinoma, is the family initiated into a genetic counseling pathway?
    - NO
    - YES

13. If YES:
    - In the same institution
    - In other institution
