# Supplementary material for: BRCA1/2 Molecular Assay for Ovarian Cancer Patients: A Survey through Italian Departments of Oncology and Molecular and Genomic Diagnostic Laboratories
Source: Diagnostics (Basel). 2019 Oct 9;9(4):146. doi: 10.3390/diagnostics9040146 (PMC6963957; doi:10.3390/diagnostics9040146)
Supplement: Supplementary file 1 [file diagnostics-09-00146-s001.zip › diagnostics-573360-Supplemental File/diagnostics-573360-Supplemental File2.pdf]

SURVEY FOR MOLECULAR BIOLOGY LABORATORIES
Mutational status assessment
# of the *BRCA1* and *BRCA 2* germline and/or somatic genes

Germline BRCA

1. In which year did the activity begin? ☐ *(integer)*

2. Which technologies are used for the complete BRCA test? *(multiple answers possible)*
   - ☐ NGS
   - ☐ Sanger
   - ☐ MLPA
   - ☐ Other *(specify)* ______

3. If you use NGS, please specify which technologies:
   - ☐ Commercial kits CE-IVD
   - ☐ Commercial kits RUO
   - ☐ Homemade pipeline

4. What is the **total number of tests carried out in 2016**? ☐ *(integer)*

5. Specify: *(integer)*

   Total number of tests performed on breast cancer patients ☐

   Total number of tests performed for patients with ovarian cancer ☐

   Total number of tests performed for patients with other tumours ☐

   Total number of tests performed on healthy subjects ☐

6. What is the total number of **complete tests** carried out in 2016? ☐ *(integer)*

7. What is the total number of **genetic tests for the family variant ALREADY KNOWN** carried out in 2016? ☐ *(integer)*

Somatic BRCA

1. Is the BRCA test performed on tumor tissue?
   - ☐ Yes
   - ☐ No.

2. If the answer is YES: it is performed on tumor tissue:
   - ☐ Paraffin wax
   - ☐ Frozen

3. Is the tumor tissue reviewed by a PATOLOGIST before the BRCA test?
   - ☐ Yes
   - ☐ No

4. In which year did the activity begin? ☐ *(integer)*

5. Which NGS technologies are used for the complete BRCA test?
   - ☐ Commercial kits CE-IVD
   - ☐ Commercial kits RUO
   - ☐ Homemade pipeline

6. What is the **total number of tests carried out in 2016**? ☐ *(integer)*

7. Specify:

   (integer)

   Total number of tests performed for patients with ovarian cancer ☐

   Total number of tests performed for patients with breast cancer ☐

   Total number of tests performed for patients with other tumours ☐

VUS

1. What criteria are used for the **classification of** variants (VUS)?
   - ☐ IARC
   - ☐ ACMG/AMP
   - ☐ Other

   (specify) ☐

2. What criteria are used for the **interpretation of** variants (VUS)?
   - ☐ ENIGMA
   - ☐ ACMG/AMP
   - ☐ Other

   (specify) ☐
